# Gastroesophageal reflux and antacid therapy in IPF: analysis from the Australia IPF Registry

**DOI:** 10.1186/s12890-019-0846-2

**Published:** 2019-05-03

**Authors:** Helen E. Jo, Tamera J. Corte, Ian Glaspole, Christopher Grainge, Peter M. A. Hopkins, Yuben Moodley, Paul N. Reynolds, Sally Chapman, E. Haydn Walters, Christopher Zappala, Heather Allan, Gregory J. Keir, Wendy A. Cooper, Annabelle M. Mahar, Samantha Ellis, Sacha Macansh, Nicole S. Goh

**Affiliations:** 1 Department of Respiratory Medicine, Royal Prince Alfred Hospital, Missenden Road, Camperdown, Sydney, NSW 2050 Australia; 2 Faculty of Medicine, University of Sydney, Sydney, NSW Australia; 3 National Health and Medical Research Council Centre of Research Excellence in Pulmonary Fibrosis, University of Sydney, Sydney, NSW Australia; 4 Department of Allergy and Respiratory Medicine, The Alfred Hospital, Melbourne, VIC Australia; 5 Faculty of Medicine, Monash University, Melbourne, VIC Australia; 6 Department of Respiratory Medicine, John Hunter Hospital, Newcastle, NSW Australia; 7 School of Medicine, University of Queensland, Brisbane, QLD Australia; 8 Queensland Lung Transplant Service, The Prince Charles Hospital, Brisbane, QLD Australia; 9 Department of Respiratory Medicine, Fiona Stanley Hospital, Perth, WA Australia; 10 Department of Respiratory Medicine, Royal Adelaide Hospital, Adelaide, SA Australia; 11 University of Tasmania, Hobart, TAS Australia; 12 Department of Thoracic Medicine, Royal Brisbane & Women’s Hospital, Brisbane, QLD Australia; 13 Lung Foundation Australia, Brisbane, QLD Australia; 14 Department of Respiratory Medicine, Princess Alexandra Hospital, Brisbane, QLD Australia; 15 Tissue pathology and Diagnostic Oncology, Royal Prince Alfred Hospital, Sydney, NSW Australia; 16 School of Medicine, Western Sydney University, Sydney, NSW Australia; 17 Department of Radiology, The Alfred Hospital, Melbourne, VIC Australia; 18 Department of Respiratory Medicine, Austin Hospital, Melbourne, VIC Australia; 19 Institute for Breathing and Sleep, Melbourne, VIC Australia

**Keywords:** Idiopathic pulmonary fibrosis, Gastroesophageal reflux disease, Antacid, Registry, Cough

## Abstract

**Background and objective:**

Gastroesophageal reflux disease (GORD) is highly prevalent in idiopathic pulmonary fibrosis (IPF) and may play a role in its pathogenesis. Recent IPF treatment guidelines suggest that all patients with IPF be considered for antacid therapy. However, emerging evidence suggests that antacid therapy does not improve IPF patient outcomes and may increase the risk of pulmonary infection.

**Methods:**

Using prospectively collected data from the Australian IPF Registry including use of antacid therapy, GORD diagnosis and GORD symptoms, the relationship of these GORD variables to survival and disease progression was assessed. The severity of GORD symptoms using the frequency scale for symptoms of GORD (FSSG) and its relationships to outcomes was also assessed for the first time in an IPF cohort.

**Results:**

Five hundred eighty-seven (86%) of the 684 patients in the Australian IPF Registry were eligible for inclusion. Patients were mostly male (69%), aged 71.0 ± 8.5 years with moderate disease (FVC 81.7 ± 21.5%; DLco 48.5 ± 16.4%). Most patients were taking antacids (*n* = 384; 65%), though fewer had a diagnosis of GORD (*n* = 243, 41.4%) and typical GORD symptoms were even less common (*n* = 171, 29.1%). The mean FSSG score was 8.39 ± 7.45 with 43% (*n* = 251) having a score > 8. Overall, there was no difference in survival or disease progression, regardless of antacid treatment, GORD diagnosis or GORD symptoms.

**Conclusions:**

Neither the use of antacid therapy nor the presence of GORD symptoms affects longer term outcomes in IPF patients. This contributes to the increasing evidence that antacid therapy may not be beneficial in IPF patients and that GORD directed therapy should be considered on an individual basis to treat the symptoms of reflux.

## Introduction

Despite recent treatment advances [1, 2], idiopathic pulmonary fibrosis (IPF) remains an irreversible fibrotic lung disease associated with a poor survival [3]. While IPF is limited to the lungs, co-morbidities are common in this population and many studies have shown that gastroesophageal reflux (GOR) is highly prevalent [4–7], may contribute to pathogenesis and is reported to be associated with a better survival [8, 9]. Gastroesophageal reflux disease (GORD) is defined as the presence of troublesome gastroesophageal reflux symptoms (at least twice a week) and/or complications including oesophageal injury [10, 11]. However, given the subjective nature of symptoms, the accurate diagnosis of GORD remains elusive. While objective testing for oesophageal injury at endoscopy can be performed, there is poor correlation with reflux symptoms. Only 39% of patients with oesophageal injury reported typical reflux symptoms in one study, and more than 60% of patients who reported reflux symptoms had no evidence of oesophageal injury [12]. Twenty four hour ambulatory pH has also been used as an objective measure of GOR, although only 25% of patients with abnormal acid reflux on testing reported typical GORD symptoms [7]. Additionally, ambulatory pH monitoring cannot detect the presence of non-acid reflux, which may have important clinical implications, further limiting the utility of this test.

Current IPF treatment guidelines make a conditional recommendation for considering antacid therapy for all IPF patients irrespective of GORD symptoms [13]. This was based on early retrospective data demonstrating improved survival [8] and slowing disease progression [14] in IPF patients on antacid therapy. Subsequent post-hoc analyses from both the pirfenidone [15, 16] and nintedanib [17] studies, have demonstrated no benefit of antacid therapy on longer term outcomes for either the placebo [15] or treatment [16] populations, calling into question this recommendation.

Using data from the Australian IPF Registry, we explored: 1) the use of antacid therapy in our IPF population; and 2) the presence of GORD symptoms and disease, on disease progression and survival. For the first time in IPF, we also utilised the frequency scale for symptoms of GORD (FSSG) which was developed to predict the presence of endoscopic features and severity of oesophagitis resulting from GORD [18]. If clinically meaningful, this questionnaire may be a simple way to predict prognosis and treatment in IPF without resorting to invasive testing.

## Methods

### Population

All participants from the Australian IPF Registry who had completed questionnaires regarding co-morbidities, treatment and reflux symptoms were included in the study. The Australian IPF Registry is a prospective national registry which was established in 2012 for patients with IPF across Australia. Demographic, questionnaire and objective investigational data are collected at baseline and six-monthly during follow up. Details of the Registry have been published previously [19]. The data analysis for this study has ethical approval from the Sydney Local Health District ethics committee (protocol number X14–0264).

### GORD related variables

In the baseline Registry questionnaire, all Registry participants are routinely asked about the following:

1. Diagnosis of GORD (Yes/No)
2. Typical GORD symptoms of heartburn, reflux or sour taste in mouth after eating (Yes/No)
3. List of current medications
4. Frequency scale for the symptoms of GORD (FSSG) (Table 1). A symptom score of > 8/48 was used to define significant symptoms in categorical analysis based on previously reported data regarding accuracy in diagnosing endoscopic oesophagitis [18].

**Table 1:** Frequency scale for the symptoms of GORD (FSSG)

	Never	Occasionally	Sometimes	Often	Always
1. Do you get heartburn?	0	1	2	3	4
**2. Does your stomach get bloated?**	0	1	2	3	4
**3. Does your stomach ever feel heavy after meals?**	0	1	2	3	4
4. Do you sometimes subconsciously rub your chest with your hand?	0	1	2	3	4
**5. Do you ever feel sick?**	0	1	2	3	4
6. Do you get heartburn after meals?	0	1	2	3	4
7. Do you have an unusual (eg burning) sensation in your throat?	0	1	2	3	4
**8. Do you feel full while eating meals?**	0	1	2	3	4
9. Do some things get stuck when you swallow?	0	1	2	3	4
10. Do you get bitter liquid (acid) coming up into your throat?	0	1	2	3	4
**11. Do you burp a lot?**	0	1	2	3	4
12. Do you get heartburn if you bend over?	0	1	2	3	4
Total Acid reflux symptoms (total = 48)					
**Total Dyspeptic (dysmotility) symptoms (total = 20)**					

Total Dyspeptic (dysmotility) symptoms are in bold

### Statistical analysis

Results are presented as mean ± standard deviation (SD), median and interquartile range (IQR) or n (%) as appropriate. Comparisons between groups were made using Student’s t test or chi squared. An unstructured, linear mixed model with random intercepts and slopes was used to calculate the annual decline in FVC % predicted. Survival analysis was performed with Cox proportional hazards and Kaplan Meier methodology. All results with a *p* value < 0.05 are reported as significant.

## Results

### Baseline characteristics

Of the 684 patients in the Registry at the time of this analysis, 587 (86%) patients had completed the baseline questionnaire. The characteristics of this population were typical for IPF with most patients being older (71.0 ± 8.5 years), mostly male (*n* = 406; 69%) and ex/current smokers (*n* = 424; 72%) (Table 2). The majority of patients were taking antacid therapy (*n* = 384; 65%) at the time of entry into the Registry, with most receiving proton pump inhibitors (PPIs) (*n* = 344, 90.0%), and fewer receiving Histamine-2 receptor antagonists (H2RA) (*n* = 10, 2.6%) and a small proportion on both PPIs and H2RAs (*n* = 30, 7.8%). Of those receiving antacid treatment, 193 patients (50%) had significant symptoms (FSSG> 8) while 191 patients (50%) reported less significant reflux symptoms (FSSG≤8). The self-reported prevalence of GORD was 41.4% (*n* = 243) but only 29.1% (*n* = 171) reported typical symptoms of “heartburn, reflux or sour taste in mouth after eating”. The mean FSSG score was 8.39/48 (SD 7.45), with 43% (*n* = 251) patients having a FSSG score > 8.

**Table 2:** Clinical characteristics and prevalence of GORD related features

	n	overall	SD or %	antacid		no antacid		*p*
	587			384	65%	203	35%	
Age	586	71.0	8.5	71.6	8.2	69.7	8.8	0.008
male	587	406	69%	258	67%	148	73%	NS
Ever smoker	587	424	72%	280	73%	144	75%	NS
BMI	450	28.9	4.8	28.8	4.5	29.0	5.4	NS
FVC (L)	462	2.6	0.8	2.6	0.77	2.6	0.76	NS
FVC (%pred)	462	81.7%	21.4%	83.6%	22.6%	78.3%	18.8%	0.009
DLco (%pred)	409	48.5%	16.4%	49.8%	16.1%	46.1%	16.6%	0.027
CPI	405	44.93	13.88	43.83	14.16	46.87	13.19	0.034
SGRQ	550	47.6	20.5	48.57	20.5	45.2	20.7	0.072
FSSG symptom score	587	8.4	7.5	9.7	7.9	5.9	5.7	< 0.001
GORD typical symptoms	587	171	29%	139	36%	32	16%	< 0.001
GORD disease	587	243	41%	208	54%	35	17%	< 0.001
FSSG > 8	587	251	43%	193	50%	58	29%	< 0.001

*BMI* Body Mass Index, *FVC* Forced Vital Capacity, *DLco* diffusion capacity for carbon monoxide, *CPI* composite physiological index, *GORD* gastroesophageal reflux disease, *SGRQ* St George’s Respiratory Questionnaire, *FSSG* frequency scale for the symptoms of GORD

### Disease progression and overall survival

During a median follow up period of 2.2 years (IQR 1.3 to 3.4 years), 240 (40.9%) patients died and 33 (5.6%) had a lung transplant.

There was no difference in the annual fall in FVC %predicted demonstrated, regardless of whether patients were receiving antacid therapy (compared to those not on therapy), or had a GORD diagnosis (compared to those without a diagnosis of GORD), or had significant GORD symptoms (FSSG> 8) (compared to those without significant GORD symptoms (FSSG ≤8) (Table 3).

**Table 3:** Annual decline in FVC% predicted by GORD variable

GORD variable	Yes (95% CI)		No (95% CI)		*p*
Antacid therapy	4.0%	3.3, 4.8%	3.7%	2.7, 4.7%	0.614
GORD diagnosis	4.6%	3.5, 5.4%	3.5%	2.7, 4.4%	0.162
Typical reflux symptoms	4.0%	3.0, 5.1%	3.7%	3.0, 4.4%	0.612
FSSG> 8	4.5%	3.5, 5.4%	3.5%	2.7, 4.4%	0.153

**Fig. 1:**
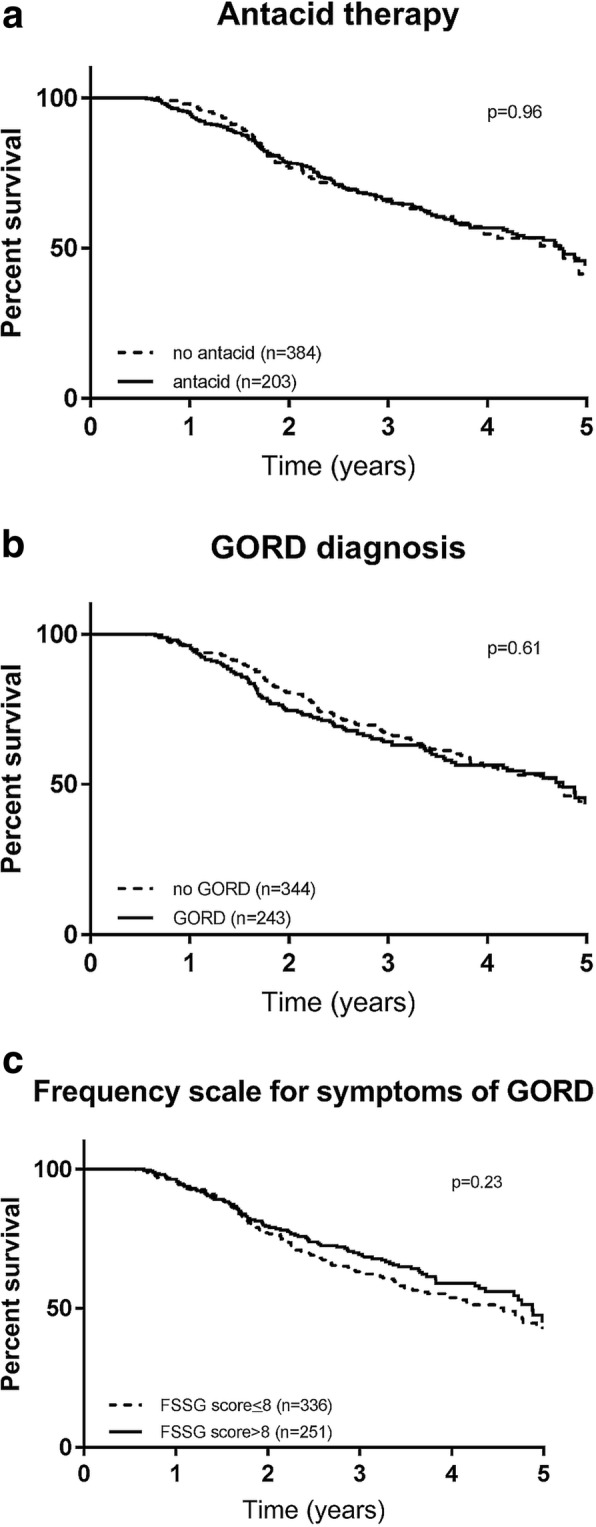
Kaplan Meier analysis for overall survival. **a**. Antacid therapy **b**. Gastroesophageal disease diagnosis **c**. Frequency scale for the symptoms of GORD (FSSG)

On univariable analysis for mortality, there was also no difference between patients who were receiving antacid treatment compared to those not receiving antacid treatment (Table 4). There was also no association between the presence of a GORD diagnosis or significant GORD symptoms with overall survival (Fig. 1).

**Table 4:** Univariable Cox analysis for Overall

	HR	95% CI		*P*
Age	1.03	1.01	1.04	0.001
male	1.34	1.01	1.79	0.043
smoker	1.39	1.03	1.87	0.032
BMI	0.95	0.92	0.98	0.002
FVC % predicted^a^	0.78	0.72	0.84	< 0.001
DLco % predicted^a^	0.56	0.5	0.63	< 0.001
GORD treatment	0.99	0.75	1.31	NS
GORD typical symptoms	0.88	0.66	1.16	NS
GORD disease	1.07	0.83	1.39	NS
FSSG score > 8	0.85	0.66	1.10	NS

^a^for every 10 unit change; *BMI* Body Mass Index, *FVC* Forced Vital Capacity, *DLco* diffusion capacity for carbon monoxide, *GORD* gastroesophageal reflux disease, *FSSG* frequency scale for the symptoms of GORD

On multivariable analysis, there was no difference in survival between antacid therapy groups HR = 1.02; 95% CI 0.72–1.43; *p* = 0.928), adjusting for age, gender, smoking, FVC %predicted and DLco %predicted. Increasing age, reducing FVC %predicted and DLco %predicted levels, were independent predictors of worse overall survival.

**Fig. 2:**
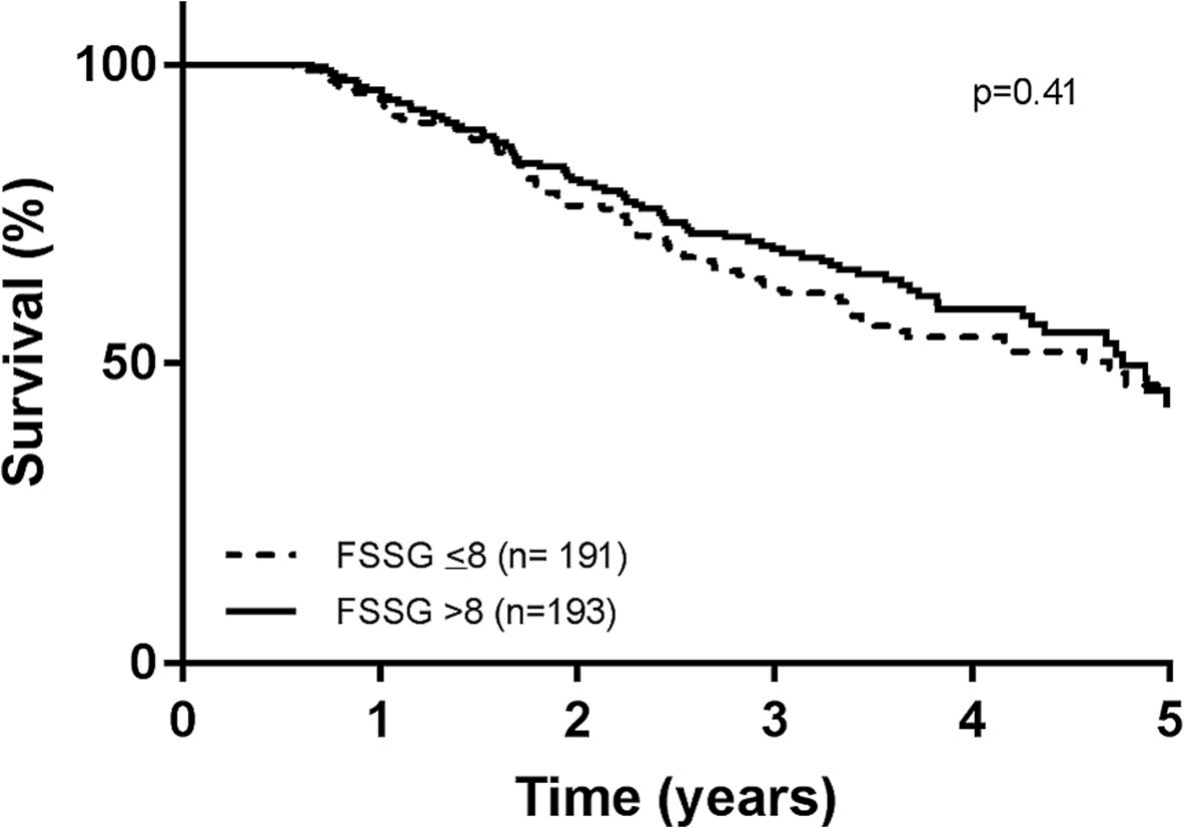
Kaplan Meier analysis for overall survival in patients on antacid therapy by presence of symptoms

In the subgroup of 384 patients receiving antacid therapy, there was also no difference in survival (HR = 0.88, 95% CI = 0.64,1.20; *p* = 0.415) between those with significant GORD symptoms (FSSG> 8), compared to those without (FSSG≤8) significant GORD symptoms (Fig. 2). There was also no difference in survival based on GORD diagnosis or typical GORD symptoms.

## Discussion

This is one of the largest retrospective cohort studies of antacid therapy in IPF and is the first study to utilise the FSSG score to assess GORD symptom severity in patients with IPF. In this analysis of real-world IPF patients from the Australian IPF Registry, treatment with antacid therapy did not have any impact on either IPF disease progression or survival, regardless of the presence of reflux symptoms. There was also no association between either the presence of typical reflux symptoms nor symptom severity with IPF disease progression or survival. In the absence of prospective, randomised controlled trials showing benefit of antacid medications in IPF, this study suggests that antacid therapy should not be recommended broadly for the treatment of IPF patients.

There has been extensive debate regarding the utility of antacid therapy in IPF. A retrospective analysis of 204 patients by Lee et al. [8] showed that reported use of antacid therapy was associated with decreased radiological fibrosis and was an independent predictor of longer survival. Patients with typical reflux symptoms (heartburn or regurgitation) as well as those with patient or physician reported GORD diagnosis, were also found to have improved survival [8]. Unlike the study by Lee et al. [8], our study of 587 IPF patients did not show any association between GORD diagnosis, reflux symptoms or antacid therapy with survival. Similar to the post-hoc analysis of the 624 patients on placebo [15] and 623 patients on pirfenidone [16], we found that there was no difference in disease progression or survival in patients on antacid therapy. This however, is in contrast to the post-hoc analysis of 242 patients from the IPFnet trials which showed patients on antacid therapy had a slower decline in FVC [14]. In a recent meta-analysis [20], treatment of GORD was associated with a reduction in IPF-related mortality but not all cause mortality. The authors report however, that the quality of evidence was low and was based on 3 studies [15, 16, 21]. Given the conditional recommendation for antacid therapy in the 2011 and 2015 IPF treatment guidelines [13], our study highlights the need for prospective randomised, controlled studies in this controversial area.

The conflicting results surrounding antacid therapy in IPF may reflect the contribution of non-acid reflux in the pathogenesis of IPF. While it has been postulated that micro-aspiration of acid results in lung injury, refluxate in GORD also contains other potentially harmful substances such as bile salts, enzymes and bacteria [22]. A study of bronchoalveolar lavage (BAL) showed an increase in pepsin and bile acids in patients with IPF (*n* = 13/16) compared to patients with other interstitial lung diseases (ILDs) (*n* = 5/20) and those with no ILD (*n* = 0/16) [6]. Animal studies have shown that acid aspiration can cause collagen deposition and disruption of parenchymal architecture [23], while others have shown parenchymal fibrosis in chronic aspiration is independent of acidity [24]. The reflux of non-acid substance may not result in symptoms nor respond to antacid therapy, while still causing lung injury and explain the lack of association between symptoms and antacid treatment with IPF outcomes.

Anti-reflux surgery would prevent the reflux of both acid and non-acid refluxate and thus a prospective, randomised controlled trial to compare the decline in FVC between surgery and non-surgery groups has been performed. In this study of 58 IPF patients, patients who had surgery had a slower decline in FVC over 48 weeks (50mLs) compared to those who did not have surgery (130mLs). However this difference did not reach statistical significance (*p* = 0.28), and has been ascribed possibly to the small sample size [25]. With a recent meta-analysis of case control studies suggesting that the association between IPF and GORD may not be directly causal but be explained by confounding factors, particularly with smoking, the recommendation to broadly treat GORD in IPF is further brought into question [26].

Another major problem facing the investigation of the relationship between GORD and IPF is the subjective nature of the GORD definition. While GORD is defined as the presence of troublesome symptoms and/or complications [11, 27], “troublesome symptoms” are subjective and whether the presence of cough in IPF fits this criteria is highly debatable [28]. In this study, we used the standardised FSSG questionnaire to characterise the severity of reflux symptoms in patients with IPF. The FSSG questionnaire was initially developed by screening 50 symptom questions to a group of 124 patients with an endoscopic diagnosis of oesophagitis. 12 questions were then selected and validated in a separate cohort of patients with and without GORD. They reported a sensitivity of 62%, a specificity of 59% and accuracy of 60% at a score of 8/48 for the presence of endoscopically visible reflux oesophagitis [18]. The relatively low accuracy of this score again reflects the subjective nature of symptoms. That said, compared to other studies where the rate of oesophagitis was 38.7% in patients who had typical symptoms [12] and 21.4% in patients with a GORD diagnosis [21], the FSSG questionnaire appeared to have greater accuracy. Improvement in endoscopic oesophagitis that were mirrored by improvements in FSSG scores is also encouraging as to the utility of this score [18]. In our study, there was no relationship between this questionnaire and IPF outcomes, suggesting that the FSSG questionnaire may be of use when considering treatment with antacid therapy for reflux oesophagitis without the need for endoscopy, but is not helpful in differentiating patients at greater risk for IPF disease progression or death.

Due to the retrospective nature of this study, there are several limitations. Firstly, the diagnosis of GORD was self-reported and may under or overestimate prevalence. For this reason, we looked at many GORD related variables to assess the impact of GORD and GORD treatment. Secondly, all antacid use was self-reported at baseline and analysed using an intention to treat approach. This approach does not reflect ongoing use nor quantify total exposure, which may be of clinical significance. In a retrospective study of 786 IPF patients, the use of PPIs for > 4 months was associated with reduced mortality whereas use for 2 or 3 months was not [21]. The introduction of categorisation by exposure duration however introduces immortal time bias and may explain some of the perceived benefit of therapy. A recent analysis by Tran et al. suggests that immortal time bias accounts for the beneficial effect of antacid therapy in many of the observational studies in IPF [29]. Thirdly, as this was not a randomised trial, it may be possible that patients who had the worst reflux were already on long-term treatment, mitigating their risk for disease progression. This may explain the fact that at baseline, patients on treatment had better FVC %predicted and DLco %predicted. There was however, no correlation between the severity of reflux symptoms in patients not on treatment and outcomes, suggesting that this is unlikely to be a key factor determining prognosis. Finally, the baseline characteristics between the treatment and non-treatment groups differed with patients on treatment tending to be older but have better lung function. Multivariable analysis adjusting for age, gender, smoking status, FVC and DLco however did not have any impact on outcomes. Despite the limitations, this study is one of the largest retrospective cohort studies of antacid therapy in IPF. It is also the first study to utilise the FSSG score, collected prospectively, in patients with IPF and shows that GORD symptom severity does not predict decline.

## Conclusion

While further, robust randomised controlled trials are still needed, this study adds weight to the gathering evidence that antacid therapy may not be beneficial in IPF patients and that reflux directed therapy should be considered on an individual basis. While the severity of reflux symptoms does not predict IPF outcomes, it may help select patients who will benefit from GORD treatment for management of symptoms and the prevention of oesophageal injury. Our study also highlights the difficulty in defining GORD and highlights the need to standardise the GORD definition used in future prospective randomised studies if evidence of treatment benefit is to be accurately assessed.

## Abbreviations

DLco: Diffusion capacity for carbon monoxide
FSSG: Frequency scale for symptoms of GERD
FVC: Forced vital capacity
GOR: Gastroesophageal reflux
GORD: Gastroesophageal reflux disease
H2RA: Histamine 2 receptor antagonists
ILD: Interstitial lung disease
IPF: Idiopathic pulmonary fibrosis
PFS: Progression free survival
PPI: Proton pump inhibitor
